# Streptococcus subpectoral abscess with shoulder pain: A rare emergency with a common symptom?

**DOI:** 10.4103/0973-6042.68419

**Published:** 2010

**Authors:** D. S. Angadi, S. Nagappa, D. Simpson, I. Morgan

**Affiliations:** Department of Trauma and Orthopaedics, New Cross Hospital, Wolverhampton, UK

Sir,

Lancefield group A beta-hemolytic streptococcus (GABHS) is known to cause a wide range of infections. However shoulder pain due to isolated subpectoral abscess caused by group A beta haemolytic streptococcus is extremely rare with only two previously reported cases in the literature.[1,2] The onset may be insidious and patient may appear well on initial presentation. The clinical course is varied but sometimes precipitous associated with high morbidity and mortality. We describe the third case of painful shoulder in a teenage boy due to subpectoral abscess caused by this organism. A 16-year-old otherwise healthy boy presented to the outpatient orthopaedic clinic with chief complaint of pain in the right shoulder. 2 weeks prior to this presentation he had an episode of sore throat for which he was treated by his general practitioner with paracetamol. A week later he noticed pain in his right shoulder. There was no history of recent infection to the right arm or hand, trauma, insect bite animal exposure, or recent travel.

On physical examination he was a well-built and well-nourished boy. Inspection revealed erythema and fullness of the right supraclavicular fossa and pectoral region. There was no bruise, scars or other lesions on the overlying skin. He had an oral temperature of 38.6°C and a pulse rate of 90/min. He had tenderness around right axilla with no palpable lymphadenopathy. External rotation and active abduction beyond 90° were painful in the right shoulder. The remainder of shoulder movements were normal.

Preliminary blood tests revealed that white blood cell count was 24300/mm^3^ with 85% neutrophils, 10% lymphocytes, 4% monocytes, 0.6% eosinophils, 0.1% basophils with erythrocyte sedimentation rate of 84 mm/hr, and C-reactive protein level of 224 mg/dl. Blood and urine cultures were obtained.

Chest and right shoulder radiographs revealed no significant abnormality.

**Figure 1:**
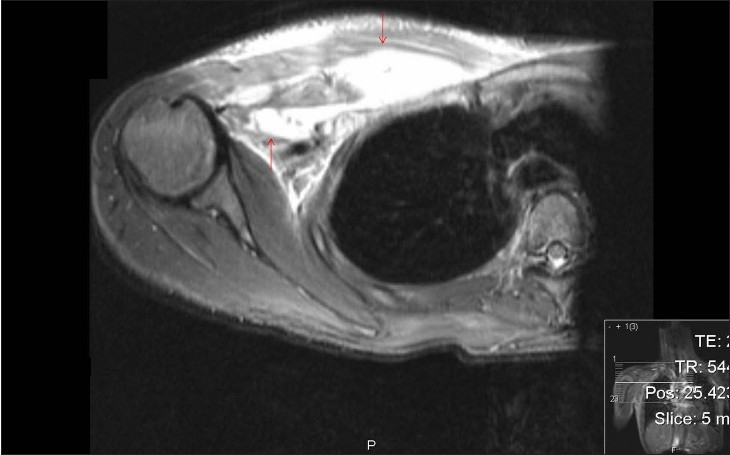
T2-weighted coronal magnetic resonance image of the right thorax. Note the abscess deep to the right pectoralis major muscle extending into the axilla (arrows)

Ultrasound scan of the right shoulder and the pectoral region showed a loculated collection deep to the pectoral muscles. Magnetic resonance imaging of the right shoulder confirmed the loculated abscess of size 3 × 3 × 9 cm lying deep to the pectoral muscles and extending into the right axilla. There was no involvement of the shoulder joint or proximal humerus [Figure 1].

Incision and drainage of the abscess was performed the following day and approximately 25 ml of pus was drained. Gram stain of the purulent material showed Gram positive cocci in chains and it was sent for aerobic, anaerobic, mycobacterial, and fungal cultures. Group A beta-hemolytic streptococci (GABHS) were isolated from microbiological culture of the abscess fluid with sensitivity to penicillin, erythromycin, and tetracycline. Blood and urine cultures remained sterile on routine and extended culture and sensitivity tests.

The patient was commenced on intravenous benzyl penicillin for a period of 48 h followed by an oral course of Penicillin V for further 2 weeks. At follow-up visit 2 weeks after discharge his operative wounds had healed well. At subsequent 3 month review, he had made good recovery from the procedure with full range of movements in the right shoulder and complete resolution of the pectoral swelling.

Lancefield group A beta-haemolytic streptococcus (GABHS) is known to cause wide range of infections with varied severity and clinical course. Commonly reported forms of infection include pharyngitis, rheumatic fever, pneumonia, streptococcal toxic shock syndrome (STSS), necrotizing fasciitis,[3] and osteomyelitis.[4]

Factors predisposing to GABHS infection include skin lesions, wounds, diabetes, nonsteroidal anti-inflammatory drug use, malignancy, and primary varicella infection.[3] Skin lesions to fingers or thumb are the most likely portal of entry. However, in almost 25% of cases with GABHS infection, there can be no particular risk factor[3] or a portal of entry.[5]

Previous authors suggested that the suppurative lymphadenitis of the deltoideopectoral lymph nodes which drain the lymphatics from the thumb and lateral half of the index finger resulted in the formation of subpectoral abscess.[2] In patients with no identified focus, the infection may originate from respiratory tract colonization leading to transient bacteraemia. Subsequently, the bacteria may get seeded at sites of minimal or unapparent local trauma.[3,5] In our patient there was history suggestive of an upper respiratory tract infection prior to the shoulder pain. However, due to the lack of trauma to either the shoulder, pectoral region or the arm it is difficult to delineate an exact aetiopathogenesis.

Subpectoral abscess due to GABHS causing shoulder symptoms is a rare presentation. Given the paucity of the literature in such cases diagnosis hinges on clinical suspicion and appropriate diagnostic studies need to be obtained early. Due to the size, loculated nature, and the extent of the abscess our patient had an incision and drainage. Once the diagnosis of subpectoral abscess was established in our patient, the functional results following treatment were remarkable.

Subpectoral abscess due to GABHS has been previously reported by Rathore MH[1] in a 22-month-old boy and Abuelreish MA[2] in a 12-year-old boy. These reports have certain interesting observations and similarities with our case. First, all the patients with this condition were males. Second, they were pyrexial with markedly raised white blood cell count and neutrophilia. Third, the patients had significant localized signs but did not look acutely ill. However, the notable differentiating feature in these cases was the age group thereby suggesting a potential wide spectrum of age groups likely to present with this condition.

The clinical course of GABHS infections is often precipitous with high mortality.[3,5] However, these patients may not appear acutely ill on initial presentation. Hence, in these cases a thorough clinical evaluation with prompt definitive treatment is vital to prevent high morbidity and mortality associated with this organism.
